# Proactive Personality and Employee Creativity: A Moderated Mediation Model of Multisource Information Exchange and LMX

**DOI:** 10.3389/fpsyg.2021.552581

**Published:** 2021-07-01

**Authors:** Aishi Zhang, Xi Li, Yuchen Guo

**Affiliations:** ^1^Business School, Nankai University, Tianjin, China; ^2^Key Lab for Behavioral Economic Science and Technology, South China Normal University, Guangzhou, China; ^3^School of Government, Sun Yat-sen University, Guangzhou, China

**Keywords:** proactive personality, incremental creativity, radical creativity, multisource information exchange, leader-member exchange (LMX), moderated mediation

## Abstract

Integrating the social perspective of creativity and the goal-regulatory process perspective of proactivity, this study investigates how proactive personality influences two forms of employee creativity-incremental creativity and radical creativity through multisource information exchange. Using a moderated mediation framework, this study suggests that leader-member exchange (LMX) moderates the positive association between proactive personality and those two forms of employee creativity. The results of this study, drawn from the sample of 500 employees and their immediate supervisors in a large state-owned company of China, support most of the hypotheses. Theoretical and practical implications of the findings, as well as the limitations of this study and the directions for future research, are discussed.

## Introduction

Employee creativity, characterized as the generation of novel and appropriate ideas by individuals and including two forms-incremental and radical creativity, is widely regarded as a powerful driver of organization innovation, survival, prosperity, and long-term development (Woodman et al., 1993; Madjar et al., 2011; Zhou and Hoever, 2014; Amabile and Pratt, 2016). Recognizing the importance of employee creativity to organizational effectiveness, scholars have devoted considerable efforts to exploring the various factors that can inspire it (Zhou and Shalley, 2003). Among these factors, proactive personality, defined as the stable and enduring behavioral inclination to take the initiative to make a constructive change of the *status quo* or create a new one, has increasingly received scholarly attention (Bateman and Crant, 1993; Fuller and Marler, 2009; Pan et al., 2018). Empirical research has confirmed that proactive employees are more likely to generate creative ideas due to their internal motivation for seeking out new approaches (Kim et al., 2009, 2010; Kim, 2019), an increased sense of responsibility to make changes (Jiang and Gu, 2015), or the emergence of positive affect activated by internal retrospective self-reflection (Li F. et al., 2019). However, this body of research has only focused on exploring the psychological mechanisms underlying the relationship between proactive personality and employee creativity, and ignores to investigate the mechanisms from a social perspective (Pan et al., 2018).

The social perspective on creativity, extended from the componential model of creativity, emphasizes that “creativity is in part a social process” (Amabile, 1983; Perry-Smith and Shalley, 2003). From this perspective, social interaction and the correspondingly social network are regarded as crucial parts of employee creativity (Perry-Smith and Shalley, 2003). Information exchange, which is embedded in the process of social interaction, can also promote the generation of creative ideas because of the change in employees’ information resources. Likewise, according to the goal-regulatory proactive process perspective of proactivity, proactive employees, with the goal of changing their environments, tend to continually interact with their surroundings to acquire information resources (Parker et al., 2010; Gong et al., 2012). And the work roles that proactive employees take on always surpass the normal job requirements (Crant, 2000; Kim et al., 2009; Pan et al., 2018), leading them to step out of their comfort zones and acquire different information, knowledge, and perspectives (Gong et al., 2012). Thus, in the present research, we integrated the social perspective of creativity and the goal-regulatory process perspective of proactivity to examine the mediating role of multisource information exchange in the relationship between proactive personality and two forms of employee creativity, where multisource information exchange refers to individuals’ conscious and deliberate attempts to exchange task information, know-how and feedback about products, techniques, and markets with others inside or outside their organizations (Bunderson and Sutcliffe, 2002; Cross and Cummings, 2004).

Although proactive personality has numerous favorable outcomes, several anecdotal evidence indicates that unanticipated consequences inevitably occur even when proactive employees take initiative (Bateman and Crant, 1993; Frese and Fay, 2001). For instance, employees often fall into the “initiative paradox” in their day-to-day work (Campbell, 2000). Specifically, the organization and its agents at each level of management want their subordinates to show initiative and exercise their own judgments regarding the potential opportunities and problems, but they also expect subordinates’ judgments and subsequent actions to be consistent with their anticipations. Thus, if the organization and its agents, such as managers or immediate supervisors, fail to clearly encourage proactivity, then the expressions of proactive personality may be suppressed or restricted (Bateman and Crant, 1993, 1999; Erdogan and Bauer, 2005). Researchers have confirmed this empirically by examining the moderating role of the close monitoring behaviors, supportive behaviors, empowering behaviors, development feedback, and visionary guidance of supervisors (Kim et al., 2010; Jiang and Gu, 2015; Pan et al., 2018; Kim, 2019). Similarly, if subordinates’ initiative is not aligned with the expectations of their organization and its agents, then the misguided efforts made by proactive subordinates can be dysfunctional and have a weaker effect (Bateman and Crant, 1999; Erdogan and Bauer, 2005; Chan, 2006). Extant research has only suggested that a closer person-organization or person-job fit results in proactive employees having higher job or career satisfaction (Erdogan and Bauer, 2005). However, whether the level of match between employees and their immediate supervisors can also help the employees receive work benefits from their proactive personality is somewhat unclear. Therefore, to address this gap, we attempted to introduce leader-member exchange (LMX) as a moderator and contended that, as the result of the cumulative effects of similarity, delegation, and performance in accordance with supervisor’s anticipation (Bauer and Green, 1996), the quality of LMX would affect employees’ benefits from proactive personality.

**FIGURE 1:**
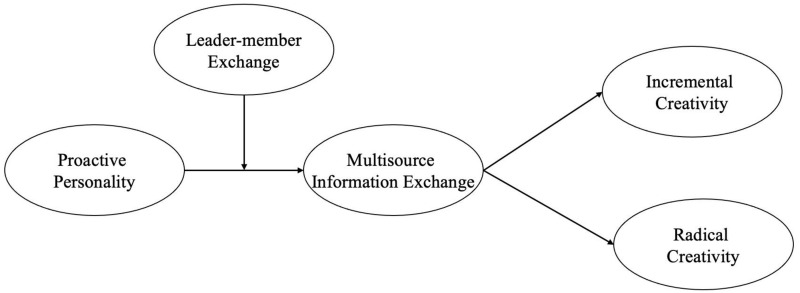
Research model.

This study makes several contributions to the literature. First, we enhance the understanding of how proactive personality fosters two forms of employee creativity. In contrast to prior research, which has explored the psychological mechanisms underlying the association between proactive personality and employee creativity, we adopt a social perspective of creativity and the goal-regulatory process perspective of proactivity to reveal that multisource information exchange is a potential underlying mechanism. The combination of the two research perspectives is useful for clarifying the proactive process directed toward creativity. Second, this study responds to the appeal of Erdogan and Bauer (2005) to investigate the moderators affecting the relationship between proactive personality and its outcomes. Drawing on LMX theory (Graen and Uhl-Bien, 1995; Bauer and Green, 1996), we propose that the interaction between proactive personality and LMX can promote employees’ multisource information exchange, in turn triggering the emergence of creative ideas. Our study not only highlights the significance of LMX for understanding the contingent creative benefits of proactive personality, but also provides a theoretical explanation for why proactive employees may require different things from their leaders than non-proactive employees. Third, our research provides helpful implications regarding how organizations can foster employee creativity by maximizing the favorable effect of proactive personality on multisource information exchange and by considering the quality of LMX. The proposed moderated mediation model has been presented in Figure 1.

## Literature Review and Hypothesis Development

### Proactive Personality and Multisource Information Exchange

With a strong desire to change their environments, proactive employees demonstrate various distinctive behaviors, such as acting in advance, persisting until meaningful change occurs, and adopting self-starting, change-oriented, future-focused, and goal-directed behaviors (Grant and Ashford, 2008; Fuller and Marler, 2009; Parker et al., 2010). These behavior can be observed “either within or beyond the boundaries of an employee’s role” (Grant and Ashford, 2008). As such, proactivity can be viewed as a dynamic process of action rather than a unique set of actions (Grant and Ashford, 2008; Parker et al., 2010), and employees’ proactive personality embodies in this particular action process of striving to manipulate the environment. The first scholars to propose the process perspective of proactivity were Grant and Ashford (2008). They suggested that this process can be applied in a series of actions through anticipation, planning and striving to have an impact. On the basis of self-regulation theory, Bindl et al. (2012) later proposed the goal-regulatory process perspective of proactivity and extended the process elements to envisagement, planning, enactment and reflection.

Unlike employees who passively adapt to their circumstances and wait for change (Bateman and Crant, 1993), proactive employees expend cognitive effort on envisioning a different future, which can bring about constructive change (Parker et al., 2010). During this envisioning process, employees may interact with others to acquire information that is useful for identifying current or future problems or opportunities (Bindl et al., 2012; Gong et al., 2012), and then establish a series of clear change-oriented goals that are compatible with their own values (Karniol and Ross, 1996). After setting proactive goals, employees enter a crucial phase—planning, which links the envisioned future to the enactment of goal-directed action through the outlining of concrete steps. In this phase, employees with proactive personality comprehensively consider all of the scenarios that they may encounter (Taylor et al., 1998; Bindl et al., 2012) and actively search for and process additional information acquired from other people to develop alternative strategies and backup plans for meeting unexpected needs (Grant and Ashford, 2008).

Subsequently, guided by their self-started goals and action plans, these employees dedicate their time and energy to realizing desired change (Bindl et al., 2012). Given that any change, no matter how small, can lead to resistance and skepticism from others (Bateman and Crant, 1999), proactive employees may honestly share the advantages and disadvantages of the strived-for change with others and patiently listen to others’ opinions and suggestions (Parker et al., 2010). Persisting in implementation can help proactive employees keep their focus on task-related interactions and stop them from becoming distracted by non-task requirements (Frese and Fay, 2001), especially when serious setbacks or failures occur. Likewise, reflection is another effective approach that proactive employees adopt to overcome obstacles. In addition to reflecting on failure events, employees with proactive personality are inclined to engage in reflective learning from successes (Li F. et al., 2019). Although reflection on past events is an internal rather than external causal attribution process, it may trigger the starting or braking mechanisms of subsequent knowledge acquisition processes (Ellis et al., 2006). For instance, proactive employees’ reflective learning from successes and failures can encourage them to seek and integrate information from others through the activation of joviality and attentiveness affect, respectively (Li F. et al., 2019).

As mentioned, proactive personality is not constrained by situational forces, and its range of action is not limited by the organizational boundaries. Thus, proactive employees may continually exchange information with various stakeholders from inside and outside their organization, such as coworkers in the same departments, immediate supervisors, colleagues in other departments, customers or distributors, suppliers, and cooperation partners outside their organization. And as the dynamic process of proactivity unfolds, the intensity and duration of multisource information exchange may increase. Thus, we hypothesized the following:

*H1*: Proactive personality is positively related to multisource information exchange.

### The Mediating Effect of Multisource Information Exchange

According to the social perspective on creativity, “creativity is in part a social process,” and social interactions and their corresponding social networks are critical for employee creativity (Perry-Smith and Shalley, 2003; Perry-Smith, 2006). This social perspective is extended from a widely cited theory of creativity called the componential model, which holds that the major building block of creativity at the individual level is individuals’ information resources in the task domain (Amabile, 1983, 1997; Amabile and Pratt, 2016). Individuals’ information resources can be created through four conversation modes between tacit and explicit information: externalization, internalization, socialization, and combination (Nonaka, 1994). The externalization and internalization modes focus on the mutual interactions between two aspects of information held by one person, whereas the socialization and combination modes capture the processes of using interpersonal interactions to combine information held by different individuals. Following this logic and adopting the social perspective of creativity, we propose that multisource information exchange, which is embedded in social interactions, can help induce creative ideas because of the change in employees’ information resources.

To our knowledge, an employee’s information resources comprise a range of interdependent information domains, with each information domain organized in a series of interrelated cognitive schemas (Mannucci and Yong, 2018), and each cognitive schema is formed by an array of interlinked information attributes (Dane, 2010). Information exchange with others performing diverse tasks beyond the organization’s boundary (e.g., distributers, suppliers, and external cooperation partners) may increase the number of information domains held by an employee, expanding the breadth of their information resources. Employees with broad information domains have more opportunities to identify the connections between different cognitive schemas and information attributes (Sosa, 2011; Haas and Ham, 2015), improving their ability of knowledge recombination. Exposure to diverse information categories enables employees to reconfigure the various categories and use the new information combinations in problems analysis and future prediction, increasing their likelihood of generating divergent solutions (Perry-Smith and Shalley, 2003; Gong et al., 2012). Moreover, for employees with multiple information domains, limited attention to each category makes their internal information structures to be loosely coupled systems (Haas and Ham, 2015; Mannucci and Yong, 2018), increasing the potential for flexible thinking and providing sufficient space for creative ideas to emerge (Dane, 2010).

While, information exchange with other employees performing similar tasks (e.g., coworkers in the same department, immediate supervisors, and colleagues in other departments) increases the number of information attributes an employee has and strengthens the corresponding correlations within each cognitive schema (Mannucci and Yong, 2018), enhancing the depth of the employee’s information resources in a given domain. This greater depth of information helps the employee leverage the linkages among cognitive schemas or information attributes in the given domain to discover how the boundaries can be feasibly expanded (Taylor and Greve, 2006). Employees with in-depth information may make the most of complex cognitive schemas to further search for, develop and extend seemingly ridiculous but promising novel ideas, and this eventually widens the ideas pool (Haas and Ham, 2015; Mannucci and Yong, 2018). Furthermore, for employees with in-depth information, focusing their attentions on a given domain enables them to sufficiently analyze the potential advantages and disadvantages of novel ideas, increasing the possibility of identifying and generating appropriate ideas (Perry-Smith and Shalley, 2003).

For creativity, there are two forms of that, namely incremental creativity and radical creativity. Incremental creativity refers to the adaptive ideas that imply few changes or minor modifications to existing products, processes, or platforms, whereas radical creativity refers to the breakthrough ideas that completely transform existing products, processes, or platforms and obsolete the old practices (Gilson and Madjar, 2011; Madjar et al., 2011; Gilson et al., 2012). Although some research addressed the distinction between two forms and investigated the potential different antecedents to them (Gilson and Madjar, 2011; Gilson et al., 2012; Jaussi and Randel, 2014; Li C. R. et al., 2019), other research found some common drivers to them. For example, positive affect at work broadens individuals’ receptivity to new knowledge and enhance their cognitive functioning, which in turn trigger the two forms of creative expression (Jaussi et al., 2017); failure feedback from supervisors promotes subordinates’ radical and incremental creativity by altering their interpretation of failures, establishing their confidence of actions, and increasing their willingness of learning (He et al., 2016); creative self-expectations mobilize individuals’ cognitive resources to identify potential opportunities and problems, search for various information or knowledge, and consider multiple alternatives, which then facilitating those two forms of employee creativity (Liu et al., 2019). As such, we hypothesized the following:

*H2a*: Multisource information exchange is positively related to incremental creativity.
*H2b*: Multisource information exchange is positively related to radical creativity.

On the basis of the presented reasoning and building upon H1, H2a and H2b, we posit that multisource information exchange mediates the relationship between proactive personality and two forms of employee creativity. Specifically, because they believe that they can make a constructive change to the environment or create a new one, proactive employees are more likely to experience the four-phase dynamic behavioral process involving envisagement, planning, enaction, and reflection. In each phase, proactive employees actively interact with others working inside and outside their organization to exchange information beneficial to their behavioral process. This multisource information exchange is advantageous because it expands not only employees’ breadth of information but also their depth of information, thereby increasing the likelihood they will generate novel and appropriate ideas in the workplace. In summary, we hypothesized the following:

*H3a*: Multisource information exchange mediates the relationship between proactive personality and incremental creativity.
*H3b*: Multisource information exchange mediates the relationship between proactive personality and radical creativity.

### The Moderating Role of LMX

In Section “The Mediating Effect of Multisource Information Exchange,” we propose that proactive personality has a positive impact on incremental and radical creativity through multisource information exchange. However, anecdotal evidence and empirical studies suggest that the boundary conditions must be considered if proactive personality is to be fully understood, because unanticipated consequences can occur even when employees try their best to be proactive (Bateman and Crant, 1993; Frese and Fay, 2001). One example is the “initiative paradox” (Campbell, 2000), a situation in which employees are expected to take initiative and make independent judgments while ensuring their proactivity and judgments are in line with the anticipations of their organization and its agents. Once trapped in this situation, employees are benefited less by proactive personality (Erdogan and Bauer, 2005). One solution to this paradox is to obtain more information from the agents of the organization to minimize the likelihood of unmatched expectations (Campbell, 2000). This is easier for in-group rather than out-group employees to achieve (Graen and Uhl-Bien, 1995; Bauer and Green, 1996). Therefore, in addition to the main effects, we hypothesized that proactive personality is more strongly associated with multisource information exchange when employees experience a higher level of LMX, which in turn triggers more creative ideas.

Several mechanisms could underlie this hypothesized moderating effect. First, proactive employees tend to envision a different future and set their own goals that go beyond their normal job requirements. Because the high-quality LMX signifies that the relationships between supervisors and subordinates are partnerships rather than formalized hierarchical relationships, employees easily recognize that proactivity is a desirable means of satisfying partners’ interests while also addressing their own interests (Bauer and Green, 1996). As close partners and trusted assistants, employees may have a better chance of attaining more negotiable and challenging work arrangements, which provide them with a platform for addressing problems and opportunities with others (Liden and Maslyn, 1998; DeRue and Wellman, 2009). Subsequently, to reciprocate their supervisors’ trust and expectations, employees may set themselves a series of challenging performance and learning goals, promoting them to learn and hence update their information, knowledge, and skills (Bezuijen et al., 2010). By contrast, if relationships between supervisors and subordinates are purely contractual because of low-quality LMX, employees focus primarily on self-interest and may not perform some discretionary behaviors that would benefit others or their organization, such as information exchange (Graen and Uhl-Bien, 1995; UhlBien and Maslyn, 2003). As “hired hands,” employees who experience low-quality LMX often receive undesirable or monotonous work assignments from supervisors, restricting their efforts to reframe events, features or processes (Kozlowski and Doherty, 1989; Dulebohn et al., 2012). Doing simply what their supervisor instructs them to do or meeting a meagre job requirement may further inhibit employees’ willingness to actively interact with others (Graen and Uhl-Bien, 1995). Additionally, given the low trust and little information received from supervisors, the best course of action for employees experiencing low-quality LMX is to set “do your best” goals (Bezuijen et al., 2010), which in turn reduce the willingness of these employees to learn from others.

Second, proactive employees are inclined to anticipate future developments and design various coping strategies and backup plans in advance to address these developments. Rooted in the investigation of role-making processes within complex organizations, LMX research has posited that the development of a differentiated vertical dyad relationship involves three exchange processes, namely role taking, role making, and role routinization (Graen et al., 1977; Bauer and Green, 1996; Liden et al., 1997; Schriesheim et al., 1999). Additionally, the quality of LMX has been reported to be the result of cumulative effects of similarity, delegation, and performance in accordance with supervisors’ anticipation (Bauer and Green, 1996). Thus, subordinates who experience high-quality LMX may share some demographic characteristics or personality traits with their supervisors, and these similarities may help the subordinates make judgments regarding the current situation and future development that are consistent with those of their supervisors (Zhang et al., 2012). Moreover, the frequent and continual interactions between performance and delegation in the role-making process enhance the subordinates’ understanding of what their supervisors want and need (Dienesch and Liden, 1986; Bauer and Green, 1996). The consistent judgment of supervisors and deep understanding of what is anticipated by supervisors may prevent blind information searching and increase the quality of information exchange, thereby improving the effectiveness of coping strategies and backup plans. Conversely, low-quality LMX may occur when major differences exist in demographic characteristics or personality traits between supervisors and subordinates in the role-taking process (Dienesch and Liden, 1986; Bauer and Green, 1996), and these differences may hamper the ability of subordinates to form shared perspectives with their supervisors (Zhang et al., 2012). Similarly, low-quality LMX may occur when subordinates perform poorly in the role-making process (Bauer and Green, 1996). Poor performance leads to less delegation, reducing the number of opportunities for subordinates and supervisors to learn about each other. Different perspectives and less understanding make subordinates waste energy on balancing perspective and promoting understanding with supervisors, reducing the amount of time they spend acquiring beneficial information from others.

Third, proactive employees tend to work toward achieving desired change, and this is always risky and can provoke resistance and skepticism from others. According to LMX theory, high-quality LMX is characterized by the exchange of valued tangible resources, such as budgetary support, additional materials, and advanced technical equipment, or critical intangible resources, such as trust, respect, mutual obligation, and information (Graen and Uhl-Bien, 1995; Bauer and Green, 1996; Liden and Maslyn, 1998; Janssen and Van Yperen, 2004). In other words, compared with employees who experience low-quality LMX, those who experience high-quality LMX find it easier to obtain supportive resources from supervisors, which can help proactive employees overcome much of the risk. This positive treatment may also boost the subordinates’ affective commitment toward their organization, resulting in a strong inclination to exchange information with others (Rhodes et al., 2001; Casimir et al., 2014; Hao et al., 2019). Furthermore, the possession of greater information resources and a stronger upward influence on supervisors gives employees experiencing high-quality LMX a more central role in advice networks (Erdogan et al., 2015). Centrality within advice networks can help employees share the advantages and disadvantages of desired change with others to reduce the amount of resistance and skepticism and also give them access to information known by others (Nahapiet and Ghoshal, 1998).

In sum, the preceding arguments suggest that the quality of LMX regulates the behavioral manifestation of employees’ proactive personality. Compared with employees who experience low-quality LMX, those who experience high-quality LMX may set themselves more challenging performance and learning goals, then increasing their willingness to learn from others. Consistent judgments regarding future development and a deep understanding of what is anticipated by supervisors reduce the degree of employees’ blindness during information searching, thus increasing the quality of information exchange. The convenience with which they can obtain supportive resources increases the employees’ affective commitment to their organization and strengthens their central position in advice networks, resulting in a greater inclination and sufficient opportunities to acquire information from others. Then, the increase in multisource information exchange caused by the interaction of proactive personality with high-quality LMX benefits the development of incremental and radical ideas. Accordingly, we hypothesized the following:

*H4a*: LMX moderates the strength of the mediated relationship between proactive personality and incremental creativity via multisource information exchange such that the path between proactive personality and incremental creativity through multisource information exchange are stronger when LMX is high than low.
*H4b*: LMX moderates the strength of the mediated relationship between proactive personality and radical creativity via multisource information exchange such that the path between proactive personality and radical creativity through multisource information exchange are stronger when LMX is high than low.

## Materials and Methods

### Samples and Procedures

We collected data from full-time employees and their immediate supervisors in a large state-owned manufacturing company, which had experienced trans-regional acquisitions and reorganizations and was undergoing a dramatic transformation of Lean-production, in Shanghai, China. It has more than 200 companies in six provinces and one municipality, and the total number of employees is about 25,000. Before the survey, we determined the sampling companies and size according to the enterprise scale and the number of staff in each company, and randomly selected the participants according to the employee rosters provided by the Human Resource Department. Six hundred dyad questionnaires were coded and sent in 32 companies. The questionnaires, including demographic variables, intrinsic motivation, creative self-efficacy, proactive personality, multisource information exchange and LMX, were completed by employees; and in the same time period, the rating forms of incremental creativity and radical creativity were filled out by the direct supervisors. In the cover letter of questionnaire, we indicated the purpose of investigation and the voluntary nature of participation and assured them the confidentiality of the data. All measures were translated from English into Chinese through widely used translation-back-translation procedure.

Five hundred matched and usable questionnaires were received, with a response rate of 83.3%. For the participating employees, 65% were males, average age was 34.5 years, the average organizational tenure was 8.17 years, and 44.4% of them had college and above degrees.

### Measures

#### Proactive Personality

We used 10-item scale from Bateman and Crant (1993) and Seibert et al. (1999) to measure it. The response options ranged from 1 (strongly disagree) to 7 (strongly agree). A sample item is “I am always looking for better ways to do things” (α = 0.82).

#### Multisource Information Exchange

We extended the original assessment from Subramaniam and Youndt (2005) to a 10-item scale to measure subordinates’ perception of the quality of information exchanges with various stakeholders (Table 1). The response options ranged from 1 (extremely low quality) to 7 (extremely high quality). A sample item is “I exchange information and knowledge with co-workers from the same department” (α = 0.89).

**TABLE 1:** Items and factor loadings for the scale of multisource information exchange.

	**Factor 1**	**Factor 2**
***Multisource information exchange***		
(1) I share information and learn from my coworkers in the same department.	0.79	
(2) I share information and learn from my immediate supervisor.	0.73	
(3) I interact and exchange ideas with my coworkers in the same department.	0.70	
(4) I interact and exchange ideas with my immediate supervisor.	0.70	
(5) I share information and learn from colleagues in other departments.	0.63	
(6) I interact and exchange ideas with colleagues in other departments.	0.60	
(7) I share information and learn from cooperation partners outside the company.		0.59
(8) I share information and learn from customers of the company.		0.84
(9) I share information and learn from suppliers of the company.		0.86
(10) I share information and learn from distributors of the company.		0.84

KMO = 0.87, χ^2^(45, *N* = 500) = 2982.45***, ***p < 0.001.

#### Leader-Member Exchange (LMX)

We applied a 7-item LMX scale from Scandura et al. (1986) to measure it. The response options ranged from 1 (strongly disagree) to 5 (strongly agree). A sample item is “My immediate supervisor understands my problems and needs” (α = 0.79).

#### Incremental Creativity and Radical Creativity

We used an 8-item scale developed by Gilson and Madjar (2011) to assess it. Each type of employee creativity had four items, and the response options ranged from 1 (strongly disagree) to 7 (strongly agree). The sample items are “Present refinement on how things are currently done within the company” (incremental), and “Present discoveries of completely new processes or products than what the company currently does” (radical), α = 0.87 and 0.89, respectively.

#### Control Variables

For control variables, we included education, organizational tenure, intrinsic motivation and creative self-efficacy as the suggestion of previous study (e.g., Madjar et al., 2011; Dong et al., 2015; Xu et al., 2018). Education was measured by the highest education received (1 = *middle school or below*, 2 = *technical or high school*, 3 = *junior college*, 4 = *undergraduate*, and 5 = *master’s degree or above*) and organizational tenure was measured by years. Intrinsic motivation was measured with a 7-item scale from Tierney et al. (1999) and Grant (2008), and creative self-efficacy was measured with a 3-item scale from Tierney and Farmer (2002). The sample items are “I enjoy engaging in analytical thinking” (α = 0.60) and “I have confidence in my ability to solve problems creatively” (α = 0.65), respectively.

## Results

### Confirmatory Factor Analysis

To test the distinctiveness among the study variables, confirmatory factor analyses were conducted using Mplus 7.0. The results revealed that the five-factor measurement model fit the data well [χ^2^(25, *N* = 500) = 66.62, *p* < 0.001, CFI = 0.98, TLI = 0.96, SRMR = 0.04, and RMSEA = 0.06]. All indicators had statistically significant factor loadings, suggesting convergent validity. As shown in Table 2, relative to the hypothesized five-factor measurement model, all alternative measurement models fit the data worse. For example, the model fit of the four-factor measurement model, in which incremental creativity and radical creativity were combined into a one factor [Δχ^2^(29, *N* = 500) = 77.92, *p* < 0.001, CFI = 0.94, TLI = 0.91, SRMR = 0.04, and RMSEA = 0.09], was slightly poorer than that of the five-factor measurement model. Similarly, the one-factor measurement model, in which all study variables were combined into one factor, showed a poor fit to the data [Δχ^2^(35, *N* = 500) = 582.88, *p* < 0.001, CFI = 0.68, TLI = 0.58, SRMR = 0.14, and RMSEA = 0.19].

**TABLE 2:** Comparison of measurement models.

**Models**	**χ^2^**	***df***	**Δχ^2^**	**CFI**	**TLI**	**SRMR**	**RMSEA**
Five-factor model	66.62***	25		0.98	0.96	0.04	0.06
Four-factor model (combining IC and RC into one factor)	144.54***	29	77.92	0.94	0.91	0.04	0.09
Three-factor model (combining PP and LMX into one factor, and IC and RC into another)	165.82***	32	99.20	0.93	0.90	0.05	0.09
Two-factor model (combining PP, LMX and QMIE into one factor, and IC and RC into another)	293.81***	34	227.19	0.86	0.82	0.07	0.12
One-factor model (combining PP, LMX, QMIE, IC and RC into one factor)	649.50***	35	582.88	0.68	0.58	0.14	0.19

*N = 500.*

CFI, comparative fit index; TLI, Tucker–Lewis index; SRMR, standardized root mean square residual; RMSEA, root mean square error of approximation. PP, proactive personality; LMX, leader-member exchange; QMIE, the quality of multisource information exchange; IC, incremental creativity; RC, radical creativity.

***p < 0.001.

### Descriptive Statistics

Table 3 presents the descriptive statistics and bivariate correlations for all study variables, as analyzed using SPSS 26.0. As expected, both of proactive personality and LMX were positively related to the quality of multisource information exchange, incremental creativity and radical creativity; additionally, the quality of multisource information exchange was positively related to incremental creativity and radical creativity. These results provided preliminary support for the hypotheses.

**TABLE 3:** Descriptive statistics and bivariate correlations.

**Variable**	**Mean**	**SD**	**1**	**2**	**3**	**4**	**5**	**6**	**7**	**8**
Education	2.50	0.94	–							
Organizational tenure	8.17	7.59	−0.03	–						
Intrinsic motivation	4.01	0.63	−0.08	−0.04	–					
Creative self-efficacy	3.82	0.64	−0.02	0.03	0.59***	–				
Proactive personality	5.21	0.79	0.07	0.03	0.38***	0.43***				
LMX	3.70	0.60	−0.05	0.03	0.38***	0.39***	0.46***			
Quality of multisource information exchange	4.69	1.02	0.13**	−0.08	0.32***	0.38***	0.32***	0.26***		
Incremental creativity	5.48	1.06	0.18***	0.03	0.16***	0.20***	0.22***	0.12**	0.24***	
Radical creativity	5.16	1.24	0.09*	0.11*	0.05	0.10*	0.15**	0.09*	0.16***	0.74***

N = 500.

*p < 0.05, **p < 0.01, ***p < 0.001.

### Hypotheses Testing

Mplus 7.0 was used to conduct the mediation assessment and the moderated mediation assessment, and the significance tests for the indirect effect were based on bias-corrected confidence intervals derived from 5,000 bootstrapped samples. As detailed in Table 4 (**Model 1**, **Model 3**, and **Model 4**), proactive personality was positively related to the quality of multisource information exchange (β = 0.21, *SE* = 0.07, *p* < 0.01), and the quality of multisource information exchange was positively correlated with incremental creativity (β = 0.15, *SE* = 0.05, *p* < 0.01) and radical creativity (β = 0.16, *SE* = 0.06, *p* < 0.01), supporting H1, H2a, and H2b. The indirect effect of proactive personality on incremental creativity (indirect effect = 0.032, CI_95__%_ = [0.007, 0.075]) and radical creativity (indirect effect = 0.034, CI_95__%_ = [0.009, 0.083]) was significant, and the direct effect (β = 0.15; β = 0.17, respectively) remained significantly. Taken together, this result supported H3a and H3b, that multisource information exchange partially mediated the association between proactive personality and two types of employee creativity-incremental creativity and radical creativity.

**TABLE 4:** Hypotheses testing.

***Variables***	***Quality of multisource information exchange***		***Incremental creativity***	***Radical creativity***
	***Model 1***	***Model 2***	***Model 3***	***Model 4***
***Constant***	1.07** (0.37)	2.24*** (0.39)	2.74*** (0.40)	3.29*** (0.44)
***Control variable***				
Education	0.14*** (0.04)	0.17*** (0.04)	0.18*** (0.05)	0.09 (0.06)
Organizational tenure	−0.01 (0.01)	−0.01 (0.01)	0.01 (0.01)	0.02** (0.01)
Intrinsic motivation	0.20* (0.09)	0.15 (0.08)	0.09 (0.09)	−0.09 (0.12)
Creative self-efficacy	0.38*** (0.09)	0.38*** (0.08)	0.11 (0.10)	0.06 (0.12)
***Independent variable***				
Proactive personality	0.21**(0.07)	0.18* (0.07)	0.15* (0.07)	0.17* (0.08)
***Moderated variable***				
Leader-member exchange (LMX)		0.15 (0.09)		
Proactive personality × LMX		0.26**(0.08)		
***Mediated variable***				
Quality of multisource information exchange			0.15**(0.05)	0.16**(0.06)
F	25.37***	21.16***	10.23***	5.03***
R^2^	0.20	0.23	0.11	0.06

*N = 500.*

*Values in parentheses are standard errors.*

Values in bold are relevant to tests of hypothesis.

*p < 0.05, **p < 0.01, ***p < 0.001.

**FIGURE 2:**
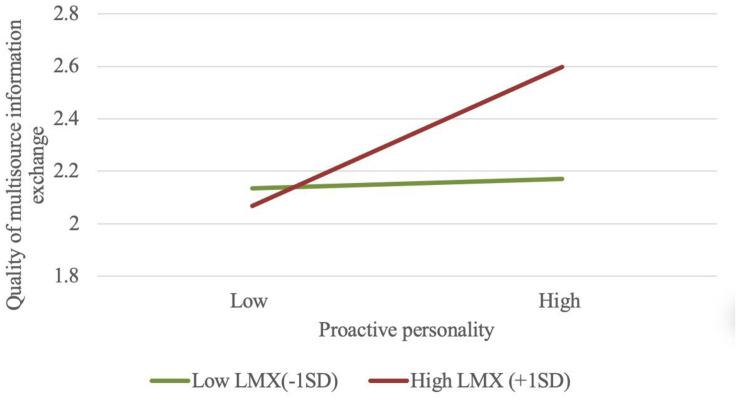
Interaction plot of proactive personality and LMX on the quality of multisource information exchange.

Tables 4, 5 present the results of moderated mediation model. As expected, the interaction of proactive personality and LMX (**Model 2**) was significant in predicting the quality of multisource information exchange (β = 0.26, *SE* = 0.08, *p* < 0.01). This is also confirmed by Figure 2, which shows that individuals with highly proactive personality exchange higher-quality information with various stakeholders when they experience high-quality LMX (simple slope = 0.335, CI_95__%_ = [0.180, 0.496]). However, the marked influence of proactive personality on the quality of multisource information exchange was not observed when LMX was low (simple slope = 0.023, CI_95__%_ = [−0.140, 0.214]). Next, we examined the conditional indirect effects of proactive personality on incremental creativity and radical creativity through the quality of multisource information exchange at three levels of LMX (1 *SD* below the mean, the mean, and 1 *SD* above the mean). The conditional indirect influence (Table 5) of proactive personality on incremental creativity and radical creativity were both significant when the level of LMX was high (indirect effect = 0.050, CI_95__%_ = [0.015, 0.105]; indirect effect = 0.054, CI_95__%_ = [0.017, 0.116], respectively), but non-significant when it was low (indirect effect = 0.004, CI_95__%_ = [−0.020, 0.038]; indirect effect = 0.004, CI_95__%_ = [−0.023, 0.042], respectively). In general, the results suggest that LMX moderates the strength of the mediated association between proactive personality and two types of employee creativity-incremental creativity and radical creativity through the quality of multisource information exchange, supporting H4a and H4b.

**TABLE 5:** Effects at different levels.

**Model**	**Level**	**P_MX_**	**P_YM_**	**Direct effect (P_YX_)**	**Indirect effect (P_YM_P_MX_)**	**Total effect (P_YX_ + P_YM_P_MX_)**
Proactive personality	LMX_low_	0.023	0.151**	0.150*	0.004	0.154*
Quality of multisource information exchange	LMX_mean_	0.179*	0.151**	0.150*	0.027	0.177**
Incremental creativity	LMX_high_	0.335***	0.151**	0.150*	0.050*	0.200**
Proactive personality	LMX_low_	0.023	0.161**	0.166*	0.004	0.170*
Quality of multisource information exchange	LMX_mean_	0.179*	0.161**	0.166*	0.029	0.195*
Radical creativity	LMX_high_	0.335***	0.161**	0.166*	0.054*	0.220*

*N = 500.*

LMX was −0.60 (i.e., 1 *SD* below the mean) and 0.60 (i.e., 1 *SD* above the mean) for low and high levels of LMX, respectively.

*p < 0.05, **p < 0.01, ***p < 0.001.

### Supplementary Test

Up to this point, we used the scale of quality perception to measure the extent of multisource information exchange and examined the mediation role of multisource information exchange in the association between proactive personality and two types of employee creativity. However, we thought it might prove worthwhile to obtain a well-rounded understanding of the effect of multisource information exchange. More specifically, we reasoned that in addition to assessing the impact of multisource information exchange quality which only represents the employees’ perception of interaction effectiveness, examining the influence of multisource information exchange frequency which describes the employees’ experience of interaction density would also prove worthwhile. For this purpose, we adopted the same extension of Subramaniam and Youndt’s (2005) measurement in our analysis and obtained responses ranged from 1 (never communicate) to 7 (communicate several times daily) to determine the frequency of multisource information exchange (α = 0.88). This frequency was discovered to be positively related to proactive personality (*r* = 0.23, *p* < 0.001), LMX (*r* = 0.25, *p* < 0.001), incremental creativity (*r* = 0.14, *p* < 0.01), and radical creativity (*r* = 0.08, *p* < 0.10).

**FIGURE 3:**
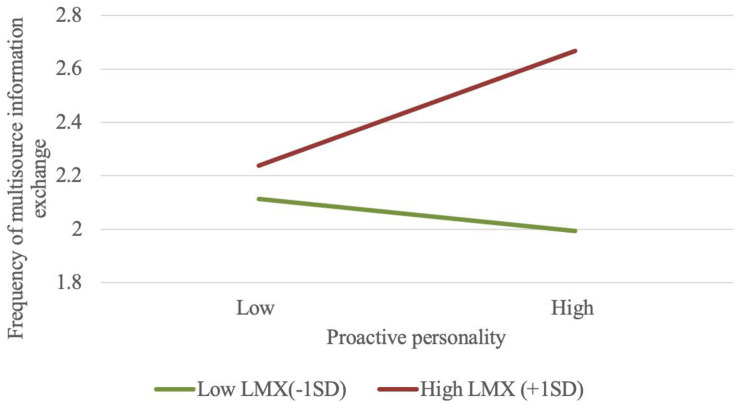
Interaction plot of proactive personality and LMX on the frequency of multisource information exchange.

The six-factor measurement model, which included both the quality and the frequency of multisource information exchange, had an unacceptable fit to the data [χ^2^(39, *N* = 500) = 320.72, *p* < 0.001, CFI = 0.89, TLI = 0.81, SRMR = 0.06, and RMSEA = 0.12]. The results of mediation analysis indicated that the quality of multisource information exchange still contributed to the bridge between proactive personality and two types of employee creativity, but the frequency of multisource information exchange was not part of this bridge. Specifically, proactive personality was positively associated with the frequency of multisource information exchange (β = 0.18, *SE* = 0.09, *p* < 0.05); however, the frequency of multisource information exchange had a non-significant negative effect on incremental creativity (β = −0.08, *SE* = 0.06, *p* = 0.20) and radical creativity (β = −0.07, *SE* = 0.07, *p* = 0.29). These results revealed that individuals with proactive personality had high inclination to engage in frequent and high-quality multisource information exchange activities, but only the high-quality multisource information exchange could increase their incremental and radical creativity. Similar to the marked influence of proactive personality-LMX interaction on the quality of multisource information exchange, this interaction had a significant positive impact on the frequency of multisource information exchange (β = 0.29, *SE* = 0.10, *p* < 0.01). Figure 3 illustrates that individuals with highly proactive personality involve in more information exchange activities with their stakeholders when they experience high-quality LMX (simple slope = 0.271, CI_95__%_ = [0.085, 0.445]); unexpectedly, however, the low level of LMX slightly and non-significantly inhibits highly proactive individuals’ information interaction (simple slope = −0.074, CI_95__%_ = [−0.263, 0.139]). Considering the non-mediated role of multisource information exchange frequency, we posited that the influence of proactive personality-LMX interaction didn’t transfer to the development of incremental and radical creative ideas through the frequency of multisource information exchange (*p* > 0.10).

## Discussion

### Theoretical Contribution

The theoretical contributions of the present study are threefold. First, this study revealed an alternative underlying mechanism (i.e., multisource information exchange) linking proactive personality and two forms of employee creativity. Previous research, although informative, only investigated the direct effect of proactive personality on employee creativity (Kim et al., 2009, 2010; Kim, 2019) and some psychological mediating factors (Jiang and Gu, 2015; Li F. et al., 2019), while ignoring the social perspective of creativity. Such an oversight prevents comprehensive knowledge from being acquired regarding the relationship between proactive personality and employee creativity, because “creativity is in part a social process” (Amabile, 1983). Moreover, developments in computer science and technology have enabled employees to conveniently communicate with individuals outside of their organization, which can give employees a new perspective and then result in more radical innovation within the organization. However, scholars have overlooked these important sources of information and focused on the impact of information exchange among individuals within a unit or organization (Gong et al., 2012). As such, the mediating role of multisource information exchange between proactive personality and incremental and radical creativity required exploration, and our study fulfills this research gap.

Second, this study highlights that the quality rather than the frequency of multisource information exchange can act as a mediating factor in the association between proactive personality and two forms of employee creativity. Social network theory suggests that compared with the strong ties, weak ties are more strongly and positively related to employee creativity (Granovetter, 1973; Perry-Smith, 2006), because employees can receive more high-quality and non-redundant information from relatively infrequent interactions. However, previous empirical research has demonstrated that a positive association exists between the quantity and the quality of information exchange in a knowledge network (Lou et al., 2013; Sedighi et al., 2016). This paradox implies that it is reasonable to return to the two basic aspects of multisource information exchange and explore their effects. Guided with the goal of constructively changing their environments, proactive employees are inclined to acquire specific information as much as possible. Nevertheless, the results of our supplementary tests, which integrated the two basic aspects in one model, demonstrate that the quality rather than the quantity of multisource information exchange is crucial for incremental and radical creativity, expanding the current understanding of the social perspective of creativity.

Third, this study sheds more light on the cause of variations obtained in earlier research on the relationship between proactive personality and employee creativity (Jiang and Gu, 2015; Pan et al., 2018; Kim, 2019). As stated, proactive personality can bring about numerous favorable outcomes, but situations in which subordinates’ initiative is not aligned with the expectations of the organization and its agents may suppress the expression of proactive personality (Bateman and Crant, 1993, 1999; Erdogan and Bauer, 2005). Therefore, the present study focused on this malalignment of expectations and provide a response to the call for research on the moderating effect of LMX on proactive personality and its outcomes (Erdogan and Bauer, 2005). Combining the goal-regulatory process perspective of proactivity and LMX theory, we argue that LMX regulates the expression of proactive personality. The results of our data analysis support this argument and further show that the effect of the proactive personality-LMX interaction on the quality of multisource information exchange may influence incremental and radical creativity in the same direction. In other words, when the quality of LMX is high, proactive employees are more likely to exchange high-quality information with various stakeholders, which in turn fosters the development of creative ideas. Therefore, by considering the role of LMX as a moderator, this study advances the efforts aimed at formulating a contingency framework for the research on proactive personality.

### Managerial Implications

Regarding insights from the findings of the present study, low-level relational factors such as the low-quality LMX are concluded to reduce the creative benefits conferred by proactive personality. Although proactive employees are inclined to take initiative to enact constructive changes to their surroundings, poor interpersonal relationships, especially with their immediate supervisors, may inhibit their willingness to make changes. Unfortunately, although organizations invest much energy and resources in the selection and employment of proactive employees (Erdogan and Bauer, 2005), they may be unaware of the critical role of interpersonal exchange relationships in the expression of proactive personality. Therefore, organizations might benefit by carrying out training programs to increase managers’ awareness of the advantages that building good relationships with employees can have in terms of proactive subordinates’ creativity. Organizations can also encourage employees to participate in various relationship seminars to develop their communication skills and ability to handle interpersonal relationship.

Our study also shows that the quality rather than the frequency of multisource information exchange can play a mediating role in the association between proactive personality and two forms of employee creativity. Some managerial strategies can be adopted to ensure high quality of multisource information exchange. For instance, organizations could encourage employees to have various high-quality interactions with individuals inside the firm. In addition, organizations could offer rewards to employees who spend time developing external connections on the weekend or while on vacation. Moreover, organizations could bring in outside experts to offer workshops that promote the breadth and depth of important project discussion and encourage employees to build connections with those outside experts.

### Limitations and Future Research Directions

This study has several limitations worthy overcoming in future research. First, the design of the study prevented us from drawing definitive conclusions regarding the causal relationships among proactive personality, multisource information exchange and two forms of employee creativity. Although predictions were based on the theory of proactive personality and employee creativity, we cannot rule out the possibility that the generation of more creative ideas motivates employees to exchange more quality information with various stakeholders and that a consequential increase in multisource information exchange fosters the development of proactive personality. Future studies may adopt a longitudinal or experimental design to test the causal relationships among these variables.

Second, considering the ratings of proactive personality, multisource information exchange and LMX from a single source cannot rule out the common method bias, so we attempted to minimize this risk by using supervisors’ ratings of employee creativity. Additionally, the results of confirmatory factor analysis supported a five-factor measurement model and indicated that all variables were distinctly differentiated. Moreover, the influence of common method bias was removed before the moderation model was analyzed (Pierce et al., 1993). Nevertheless, it would be better in the future to measure employee creativity from multiple sources, such as their subordinates and peers, and to then compare the different impacts that these groups have on creativity.

Third, this study only examined proactive personality and its outcomes at the individual level. However, the amount of attention paid to the influence of team personality composition on team outcomes has increased recently (Chiu et al., 2016). For instance, a meta-analysis of research on proactive personality conducted by Fuller and Marler (2009) indicated that the team proactive personality can also benefit the team performance and creativity. Completely different from the proactive personality at the individual level, team proactive personality represents a team’s composition in terms of proactive personality (Wang et al., 2017). The high team proactive personality means that taking initiative to change the environment is regarded as a shared trait or norm among team members. This shared norm motivates all team members to engage in numerous boundary-spanning behaviors, such as multisource information exchange, which eventually improve team performance and creativity. In the future, researchers may investigate the topic from this perspective to determine the benefits of proactive personality at levels beyond the individual level.

Fourth, collecting data from a single organization in a single cultural context brings into question the generalizability of our findings. Scholars may replicate this study in a cross-cultural context involving multiple organization and job types. Moreover, our study considered only one relational variable, LMX, as a moderator, it may be worthwhile to include personal factors (e.g., risk propensity and learning orientation) and situational variables (e.g., LMX differentiation and abusive supervision) as moderators in the association between proactive personality and employee creativity.

## Data Availability Statement

The raw data supporting the conclusions of this article will be made available by the authors, without undue reservation.

## Ethics Statement

The studies involving human participants were reviewed and approved by Ethics Review Committee at the School of Economics and Management, South China Normal University. Written informed consent to participate in this study was provided by the participants.

## Author Contributions

AZ was in charge of data collection and wrote the main part of the manuscript. XL was participated in the design of this study as well as writing-review and editing. YG was collected important background information and drafted the first version of literature review. All authors have read and approved the final manuscript.

## Conflict of Interest

The authors declare that the research was conducted in the absence of any commercial or financial relationships that could be construed as a potential conflict of interest.
